# Deriving the Characteristic Scale for Effectively Monitoring Heavy Metal Stress in Rice by Assimilation of GF-1 Data with the WOFOST Model

**DOI:** 10.3390/s16030340

**Published:** 2016-03-07

**Authors:** Zhi Huang, Xiangnan Liu, Ming Jin, Chao Ding, Jiale Jiang, Ling Wu

**Affiliations:** 1 School of Information Engineering, China University of Geosciences, 100083 Beijing, China; zhihuang@cugb.edu.cn (Z.H.); jinming@cugb.edu.cn (M.J.); dc@cugb.edu.cn (C.D.); jjl90@163.com (J.J.); 2 Institute of Remote Sensing and GIS, Peking University, 5 Yiheyuan Road, 100871 Beijing, China; wl_19830807@sohu.com

**Keywords:** characteristic scale, heavy metal stress, GF-1, rice, data assimilation

## Abstract

Accurate monitoring of heavy metal stress in crops is of great importance to assure agricultural productivity and food security, and remote sensing is an effective tool to address this problem. However, given that Earth observation instruments provide data at multiple scales, the choice of scale for use in such monitoring is challenging. This study focused on identifying the characteristic scale for effectively monitoring heavy metal stress in rice using the dry weight of roots (WRT) as the representative characteristic, which was obtained by assimilation of GF-1 data with the World Food Studies (WOFOST) model. We explored and quantified the effect of the important state variable LAI (leaf area index) at various spatial scales on the simulated rice WRT to find the critical scale for heavy metal stress monitoring using the statistical characteristics. Furthermore, a ratio analysis based on the varied heavy metal stress levels was conducted to identify the characteristic scale. Results indicated that the critical threshold for investigating the rice WRT in monitoring studies of heavy metal stress was larger than 64 m but smaller than 256 m. This finding represents a useful guideline for choosing the most appropriate imagery.

## 1. Introduction

Nowadays, soil heavy metal pollution has become of great concern to the public given increased release of metals from industrial processes and consequential food security contamination incidents; heavy metals are highly persistent, non-biodegradable, and toxic, and they can be harmful to human health when they bio-accumulate in plants and animals or bio-magnify in the food chain [1,2,3,4,5]. This underlines the need for the development of accurate and effective methods to monitor heavy metal stress in agricultural crops grown over large areas. To monitor heavy metal stress, it is necessary to know both what are the key characteristics of the contaminated crop and how can we successfully capture those characteristics accurately. Compared to traditional ground-based methods to monitor heavy metal stress, remote sensing is a valuable resource because it can improve our monitoring capabilities by allowing us to simultaneously monitor relatively large areas quickly in a non-destructive fashion while integrating the data with those from a field-based approach [6,7,8].

There is an increasing variety of satellite sensor data available, which generally possess spatial resolutions ranging from tens of meters to kilometers; thus, investigators have to make a choice about which datasets are the most appropriate to use. Typically, the choice of scale for heavy metal stress monitoring is intuitive, and the investigator selects what data happen to be readily available under some circumstances, which may be inappropriate and lead to invalid results [9,10]. Meanwhile, with the increasing availability of diverse spatial scales, the factors that need to be considered have become more complicated and diversified [11]. Therefore, accurate monitoring of heavy metal stress in crops should be conditioned on choosing the appropriate scale at which the targeted characteristic can be fully investigated. We tentatively refer to this problem as the characteristic scale for heavy metal stress monitoring. The characteristic scale is a complicated notion for which there is no single definition [12,13]. As first explained by Quattrochi [12], the characteristic scale defines the targeted processes spatial intervals and specifies the requirements for remote sensing data used in a given application. Thus, there are two aspects of the characteristic scale that have to be addressed, namely, the target information and observation approach.

The first priority is to properly define the characteristics of the object under investigation, and in the case of heavy metal stress, this could be interpreted as finding an appropriate indicator in contaminated crops to provide the “most” representative observations. Determining the optimal characteristics for contaminated crop would both improve the efficiency of data collection and the accuracy of heavy metal stress assessments. Previous studies have demonstrated that the concentrations of metals in different fractions of rice show the following sequence of priority: root > stem > leaf > storage organ; that is to say, the accumulation of heavy metals in rice’s different parts emerges as a bottom-up declining trend, *i.e*., plant parts near the soil typically contain the highest concentrations [14,15]. Compared to other changes in the biochemical characteristics of rice suffering from heavy metal stress, such as variations in the pigment and cell structure, roots are the first and direct underground part to be contaminated by excessive amounts of heavy metals, and thus, the roots likely have strong absorption and accumulation capacities [16,17,18,19,20]. Meanwhile, the dry weight of roots (WRT) is one of the most significant parameters used to represent the respiration efficiency and moisture absorption situation of roots. Among the same lines, several studies have shown that WRT data are applicable for assessing heavy metal stress in rice tissues dynamically and quantitatively via continuous simulation of WRT with the assimilation of remote sensing data and employment of the World Food Study (WOFOST) model [21,22]. But it is worth mentioning that certain factors would affect the roots growth in rice, such as the meteorological conditions, salinization and the sodium content in the rooting zone, high ground water level and so on. Therefore, WRT could be considered as a representative indicator of heavy metal stress in rice only if the rice roots are not influenced by the factors mentioned above.

The second problem in regard to the characteristic scale relates to the utilization of the remote sensing data from the sensors with the finest scales for investigating the response characteristics of the target [23,24]. Here, we may take the characteristic scale as the optimal spatial scale for the specific application, which requires fundamental comprehension of scale issues, and numerous investigators have addressed this issue [25,26,27,28,29,30,31,32]. Ideally, there is a scale or a relatively narrow range of scales that can offer the best observations [33], and the choice of the appropriate scale depends on the information desired, the spatial structure of the scene itself, and the methods used to obtain the information from the imagery [9,34,35,36]. Rahman *et al.* [37] investigated the effects of scale in vegetation characterization with hyperspectral imagery and provided recommendations for the suitable spatial scale for retaining most of the characteristic spatial variation. Duveiller and Defourny [38] defined the explicit demand for crop area estimation and crop growth monitoring and presented a conceptual framework that considers both the pixel size and purity when underlining the spatial structures of interest in existing remote sensing systems. Tran *et al.* [39] dealt with the optimal spatial scale by taking into consideration urban object characteristics and urban structures for automatic detection from remotely sensed imagery by using the local variance method and fractal dimensions. Therefore, one can conclude that the optimal spatial scale can be selected in regard to the particular characteristic of the observed phenomena under study. However, specific investigations of the appropriate spatial scale to use in the research field of heavy metal stress monitoring in rice have rarely been conducted. Previous studies have mainly focused on the use of remotely sensed data for the study of heavy metal stress varying in complexity at a given scale [8,40]. However, with the development of diverse satellite data products, the requirements for identifying the optimal scale for heavy metal stress monitoring need to realize that achieving the best observations possible is of great importance.

The objective of this paper was to identify the optimal characteristic scale for monitoring heavy metal stress in rice in the Xiangjiang watershed study area. The WRT was considered as the representative indicator to determine the most appropriate characteristic scale. We compared and examined the performance of different spatial scales on the simulated rice WRT based on the assimilation of remote sensing data with the WOFOST model. Additionally, the assessment efficiency of different spatial scales was addressed by using statistical analyses. Finally, we conducted a qualitative ratio analysis to identify the optimal characteristic scale for the purpose of realizing better observations. These findings are expected to assist researchers and agricultural managers in finding the appropriate scale to use for providing cost effective and reliable heavy metal stress monitoring data for rice, and they may also be applicable as guidelines for selecting spatial scales in other applications.

## 2. Study Area and Datasets

### 2.1. Study Area

This research was performed in Zhuzhou (112°17′–114°07′E, 26°03′–28°01′N), an old industrial base and famous high food production area that is located in the eastern region of the Hunan Province, China. Many areas in this site are suffering from heavy metal contamination caused by industrial pollutants. Zhuzhou has a subtropical monsoon climate with an average annual air temperature of approximately 16–18 °C, and the annual precipitation amount is 1361.6 mm. The main soil type in the study area is red soil with sufficient organic matter (2%–3%). Rice is the dominant agricultural crop grown, and the major type of rice grown in this area is Boyou 9083.

**Figure 1:**
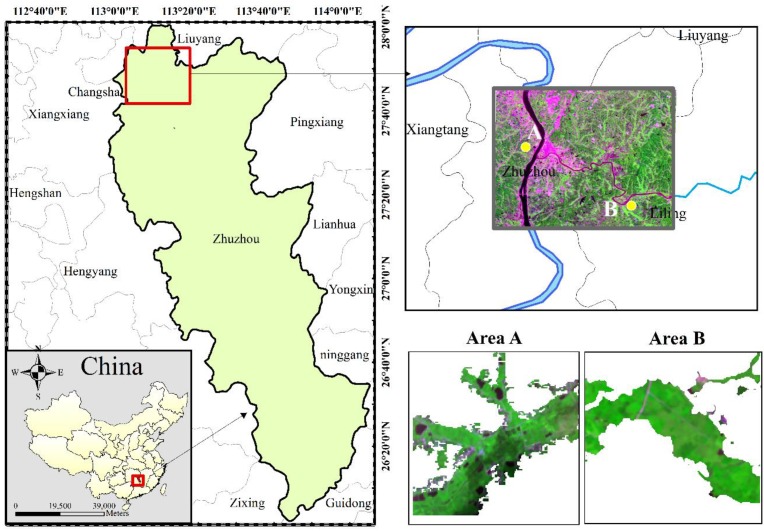
Location of the study area and the GF-1 image used in this study. GF-1 image parameters (RGB = G (0.52–0.59 μm), NIR (0.77–0.89 μm), and R (0.63–0.69 μm)) of the study areas in Zhuzhou, Hunan Province, China.

Two rice fields were selected as experimental fields (Figure 1), and each field site was 1.28 km × 1.28 km in size. Compared with Area B, Area A is more heavily polluted. Area A is located near the Xiangjiang River, which is one of the most polluted rivers in China, and Area B is adjacent to the Lujiang River, which is a tributary of the Xiangjiang River. Heavy metals (Cd, Pb, and Hg) of soil in the two study areas were both higher than background levels, and the contamination levels in Area A and Area B were categorized at the severe stress level and light stress level, respectively, based on the mean red soil of top layer and rice concentrations (Table 1). It is important to note that heavy metal stress is non-point pollution. Generally, the content of soil nutrients and the soil texture has the characteristic of spatial variability, which has a direct impact on the migration and fixation of the heavy metals in soil, and then influence the absorption and enrichment of heavy metals in rice tissues. However, the intensive planting pattern in this rice paddies makes the impact of these factors smaller. Furthermore, the rice planted in this region was cultivated and irrigated adequately to avoid other unwanted stress caused by other environmental factors such as the nutrient deficiency, pests, weeds and so on.

### 2.2. Data Preparation

The datasets collected included remote sensing data, field data, and meteorological data. All the datasets were collected from June to September 2014.

The first series of Chinese High Resolution Earth Observation System, GaoFen-1 (GF-1, meaning ‘‘high resolution’’ in Chinese) images were adopted as remote sensing data in this study. The GF-1 satellite is equipped with two 2-m panchromatic/8-m multispectral cameras and four 16-m wide-field imagers (WFI); the temporal resolution of GF-1 is 4 d. There were three 8-m multispectral GF-1 images available for the rice growth period at three acquisition dates for 2014 applicable to this study; these included ones taken on 15 June (the time is expressed as the day of the year (DOY); 15 June is thus called DOY 166), 18 July (DOY 199), and 28 August (DOY 240). These GF-1 images consisted of the following four channels: band 1: 0.45–0.52 μm (blue waveband), band 2: 0.52–0.59 μm (green waveband), band 3: 0.63–0.69 μm (red waveband), and band 4: 0.77–0.89 μm (near-infrared waveband). All the 8-m multispectral GF-1 data were radiometrically calibrated and then processed to surface reflectance through atmospheric correction using the Fast Line-of-Sight Atmospheric Analysis of Spectral Hyper-cubes (FLAASH) model. Geometric correction was performed to co-register all the images to a single coordinate system without changing their pixel resolution through the use of rational polynomial coefficient (RPC) ortho-rectification with a digital elevation model (DEM) file; all of these operations were performed in ENVI 5.1.

The field data including soil and rice parameters were collected quasi simultaneously with the acquisition of the GF-1 images. In each area, 40 sets of uniformly distributed sample data were measured using a global positioning system (GPS), and each sampling plot contained four subplots (8 m × 8 m); the average value of the four subplots was reported as the representative value for the corresponding sample plot. Rice leaf area index (LAI) values in two rice paddies were measured vertically using a botanical canopy analyzer (AccuPAR model LP-80) at every acquisition date. A SPAD-502 portable chlorophyll meter (Minolta Corporation, Ramsey, NJ, USA) was used to measure the chlorophyll content. Rice plants were selected in each subplot to obtain the WRT, which was measured by an electronic balance following a series of operations including washing and drying; then, the rice plants and 30 grams of red soil of the top layer from each subplot were air-dried at room temperature for a period of times and put into sealed plastic bags for heavy metal measurements. Heavy metal concentrations (Cd, Hg, Pb, and As) in soil and rice were analyzed at the Chinese Academy of Agricultural Sciences using an atomic absorption spectrophotometer (AAS) (Spectr AA 110/220, Varian, Palo Alto, CA, USA).

The meteorological data included air temperature (*T*) and solar radiation. The daily maximum and minimum temperature were collected from the Zhuzhou weather station in 2014. The data were used as important input parameters for the crop growth model.

## 3. Methods

**Figure 2:**
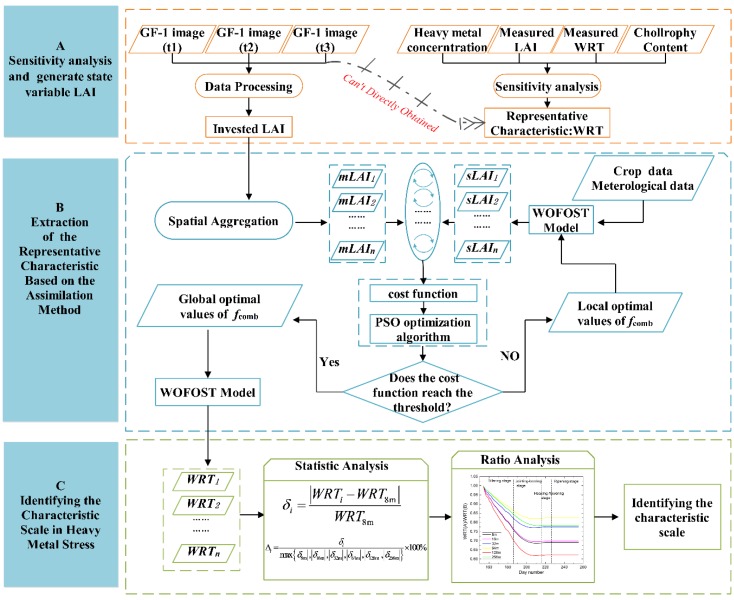
General flow chart for identifying the characteristic scale in rice heavy metal stress monitoring based on the RS-WOFOST framework.

While WRT data for rice were selected as the most representative indicator of heavy metal stress in this study, such data were difficult to acquire directly. Thus a model was employed. The Remote Sensing-World Food Studies (RS-WOFOST) framework can simulate WRT data continuously, and meanwhile, LAI is a state variable that can be obtained directly from remote sensing imagery; LAI was chosen as target observation data during the assimilation process. We up-scaled it to different spatial scales and then assimilated the series of LAI images to the WOFOST model. Finally, various statistics indicators and a ratio analysis were conducted on the simulated WRT to select the critical spatial scale for heavy metal stress monitoring in rice crops (Figure 2).

### 3.1. Retrieval and Spatial Aggregation of LAI from GF-1 Data

**Figure 3:**
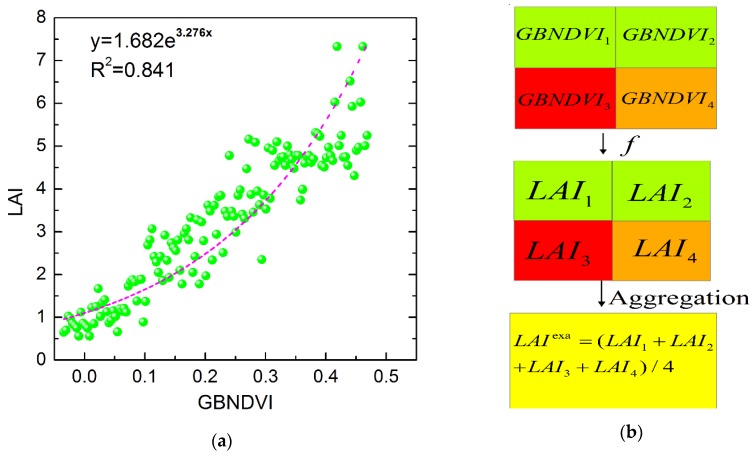
(**a**) Statistical model between the green–blue normalized difference vegetation index (GBNDVI) derived from GF-1 image data and *in situ* measured leaf area index (LAI); (**b**) Schematic flowchart of the upscaling methods for LAI estimation; f is an empirical transfer function relating to the LAI and vegetation index GBNDVI*_i_*. GBNDVI*_i_* (*i* = 1, 2, 3, 4) is the high spatial resolution GBNDVI data; LAI*_i_* is the corresponding LAI values; LAI^exa^, which is defined as the average of the LAI*_i_* (*i* = 1, 2, 3, 4), is the exact LAI value at the coarse spatial resolution.

The LAI obtained from GF-1 data is an important state variable in the data assimilation procedure as it represents the capacity of plants to capture light and assimilate carbon. Accurate inversion of LAI in the canopy is of great importance for crop growth monitoring. A great many methods for retrieving LAI from remotely sensed data have been developed in previous studies, among which the normalized difference vegetation index (NDVI) is the earliest and most widely used method. However, the most obvious disadvantage of NDVI is the saturation phenomenon in which the sensitivity of NDVI to LAI seems to decline when the LAI value is higher than the critical value (2 or 3). To resolve the saturation problem of NDVI, some researchers have explored comparisons to different modified indices based on NDVI (e.g., blue NDVI (BNDVI), green NDVI (GNDVI), green–blue NDVI (GBNDVI), and red–blue NDVI (RBNDVI)); these studies found that GBNDVI has the highest precision for retrieving LAI data [41,42]. Therefore, GBNDVI was considered as the vegetation index for the inversion of LAI here. The calculation of the GBNDVI is described in Equation (1) below, and the relationship between the measured LAI and GBNDVI computed from the GF-1 images was determined to have an exponential form, with a determination coefficient (*R*^2^) equal to 0.841 (Figure 3a):

(1) $$GBNDVI=\frac{NIR-(G+B)}{NIR+G+B}$$

(2) $$f:y=1.682e^{3.276x}$$

where *NIR* (near infrared), *G*, and *B* correspond to the reflectance in the GF-1 band 4, band 2, and band 1, respectively; x is the value of GBNDVI and y is the value of LAI. GBNDVI images at the three dates of analysis were calculated according to the GF-1 images, and the LAI images of all of the acquisition dates were obtained using Equation (2).

In order to explore the influence of different spatial scales on monitoring results for heavy metal stress in rice derived from remote sensing data, we used LAI information at six spatial scales (8 m, 16 m, 32 m, 64 m, 128 m, and 256 m). The original spatial resolution of the LAI dataset was 8 m, and the LAI images were aggregated into scales of 16 m, 32 m, 64 m, 128 m, and 256 m. There are two upscaling methods available for LAI estimates, which have been described in a previous study [43]. We adopted the method that first applies Equation (2) to the vegetation index at a high spatial resolution to calculate the corresponding LAI value and then aggregates the LAI results to obtain the exact value of LAI at a coarse spatial resolution (Figure 3b). The aggregation method applied to the LAI images of the three periods of rice growth for the two study areas involved the pixel aggregate method, which is packaged in the resize data tool in ENVI 5.1.

### 3.2. Extraction of WRT Based on the Assimilation Method

The crop growth model WOFOST is a mechanistic process-based model that simulates the allocation conditions for different parts of crop (such as leaves, stems, and storage organs) quantitatively and describes the plant growth with a time of one day based on the growth-driving and controlling processes. The model can acquire the root information easily by simulating the crop growth parameters and can be implemented in two different modes: (1) The potential growth mode, in which the solar radiation and temperature are the main factors limiting the crop growth; (2) The water-limited mode. In this study, we used the potential mode.

In order to calibrate the WOFOST model for simulating the potential growth of rice in our study areas, the crop parameters related to the rice variety (Boyou 9083), such as specific leaf area, dry matter distribution coefficient, net photosynthetic rate and the meteorological conditions (for example, the accumulated temperature) were adjusted to be constant with the measured data. Detail calibrated WOFOST parameters were reported previously [21]. Previous studies have demonstrated that the photosynthetic rate can be restrained and the dry matter of plants decreases when crops suffers from heavy metals stress, although these effects may not necessarily happen synchronously [44]. Therefore, the combining stress factor $f_{comb}$ (Equation (5)) incorporated the stress factor of the daily total gross assimilation of CO_2_ $f_{g}$ (Equation (3)), and the stress factor of the carbohydrate-to-dry matter conversion coefficient $f_{c}$ (Equation (4)) was first embedded into the WOFOST model in order to implement temporal dynamic monitoring of heavy metal stress in rice and simulate WRT more accurately; the relevant equations are as follows:

(3) $$E_{gs}(t)=f_{g}\times E_{gp}(t)\;,f_{g}\in (0.65,1)$$

(4) $$E_{cs}(t)=f_{c}\times E_{cp}(t)\;,f_{c}\in (0.65,1)$$

(5) $$f_{comb}=f_{g}\times f_{c}$$

where E_gs_(*t*) and E_cs_(*t*) are the daily total gross assimilation and carbohydrate-to-dry matter conversion coefficients under stressed growth conditions at time t, respectively; E_gp_(*t*) and E_cp_(*t*) are the corresponding parameters under the potential growth level.

Then, to find an optimal value of the stress factor, the particle swarm optimization (PSO) algorithm, which was designed by [45], was applied to minimize the value of the cost function *C* by iterating the initial parameters of the WOFOST model. The stress factor was constantly adjusted until the temporal behavior of the simulated LAI reached the best agreement with the LAI retrieved from GF-1 data. We used three dates for the GF-1 LAI datasets at six different spatial scales in this study. The expression of cost function *C* is shown as follows:

(6) $$C=\sqrt{\frac{1}{N}\sum_{i=1}^{N}\sum_{j=1}^{M}{\left(LAI_{mj(ti)}-LAI_{sj(ti)}\right)}^{2}}$$

where *N* is the total number of available GF-1 images across the entire growth period of rice, which was equal to 3. *M* represents the total number of the LAI images at different scales, and the value was 6 in this study (8 m, 16 m, 32 m, 64 m, 128 m, and 256 m). $LAI_{mj(ti)}$ is the value of LAI inverted from the j m GF-1 image on a particular day ti, and $LAI_{sj(ti)}$ represents the value of the LAI simulated by the WOFOST model on the corresponding day and spatial scale. As shown in Figure 2, when the cost function reaches the given threshold value for its minimum value, the WOFOST-simulated WRT using the optimized parameter set is the final assimilated WRT. A detailed description of the improved WOFOST model and the PSO assimilation algorithm was compiled previously [21,22].

### 3.3. Characteristic Spatial Scales Analysis

To quantify the impact of spatial scales on WRT information used for monitoring heavy metal stress in rice, we used the WRT extracts at the origin scale (8 m) as the base for comparisons. The spatial scale at which WRT characteristics (e.g., mean and variability measures) demonstrated significant changes from the base was considered to be the critical spatial scale. To find the critical scale, we calculated the absolute relative change of a WRT metric $\delta_{i}$ as

(7) $$\delta_{i}=\frac{\left|WRT_{i}-WRT_{8m}\right|}{WRT_{8m}}$$

where $WRT_{i}$ represents the mean, minimum, maximum, or standard deviation of WRT at the respective spatial scale (*i* = 8 m, 16 m, 32 m, 64 m, 128 m, and 256 m).

To facilitate comparisons across different statistics, we normalized $\delta_{i}$ by

(8) $$\Delta_{i}=\frac{\delta_{i}}{\max\left\{\left|\delta_{8m}\right|,\left|\delta_{16m}\right|,\left|\delta_{32m}\right|,\left|\delta_{64m}\right|,\left|\delta_{128m}\right|,\left|\delta_{256m}\right|\right\}}\times 100\%$$

Moreover, the ratio of WRT between the two study areas (A and B) with different stress levels was selected as an important indicator to reveal the assessment efficiency for heavy metal stress. We assumed that the value of WRT in Area A with the light stress level was the normal value; obviously, smaller ratios of WRT reflect large WRT differences in these two study areas; that is, smaller ratios indicate that there is relatively more pollution in Area B, and meanwhile, the pollution differences in the two study areas will be easier to monitor. The values of the ratios for the WRT (WRT(A)/WRT(B)) can also be used in qualitative verification analyses of the characteristic scales. Here, we present the formula for the calculation of the ratio as follows:

(9) $$Ratio_{\left(A/B\right)}=\frac{\frac{1}{m}\sum_{m=1}^{m}WRT_{A_{s}}}{\frac{1}{n}\sum_{n=1}^{n}WRT_{B_{s}}}$$

where $Ratio_{\left(A/B\right)}$ represents the ratio of WRT in the two different areas. m and n are the pixel size numbers of the study areas A and B at the spatial scale s (where s refers to 8 m, 16 m, 32 m, 64 m, 128 m, and 256 m), respectively. $WRT_{A_{s}}$ and $WRT_{B_{s}}$ represent the WRT obtained for the s scale of the study areas A and B, respectively.

## 4. Results

### 4.1. Sensitivity Analysis of Different Characteristics during Heavy Metal Stress

**Figure 4:**
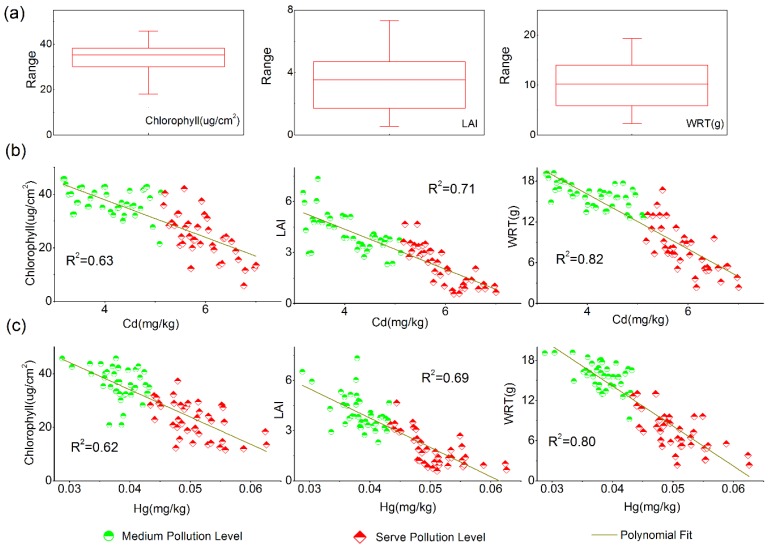
(**a**) Statistics for chlorophyll (μg·cm^−2^), leaf area index (LAI), and dry weight of roots (WRT) (g) from field measurements; (**b**) The correlation between Cd concentrations and the physiological data for chlorophyll concentrations, LAI, and WRT per rice plant; (**c**) The correlation between Hg concentrations and the physiological data for chlorophyll concentrations, LAI, and WRT per rice plant.

Different characteristics in rice presented different responses under heavy metal stress. Figure 4 shows the Hg and Cd concentration data and the corresponding characteristics (LAI, WRT, and chlorophyll content) for the sensitivity simulation and the models fitted. The boxplots of the field-collected underground, canopy structural, and functional state parameters data illustrate the response of the crops under heavy metal stress (Figure 4a). The chlorophyll content ranged from 5.85 to 45.82 μg·cm^−2^, and 90.8% of the samples had values of 11.60–40.00 μg·cm^−2^; meanwhile, the LAI (mean: 3.09, standard deviation: 1.43) and WRT (mean: 12.12 g, standard deviation: 4.70 g) maintained relatively low and steady values. In order to investigate the correlations between the biological factors (the concentrations of chlorophyll, LAI, and WRT) and the Hg and Cd heavy metal concentrations, various regression models were tried; ultimately, quadratic models were adopted because they had comparative high *R*^2^ values. As exhibited in Figure 4b,c, the *R*^2^ values between the heavy metals and the different biological parameters were similar, and the three biological characteristics were negatively correlated to the concentrations of Hg and Cd. These results also demonstrate that the best performance in regard to the sensitivity of the different biological characteristics to heavy metal stress was the underground state parameter WRT. The R² values between WRT and the concentrations of Hg and Cd were 0.82 and 0.80, respectively, and these values were greater than those for the other two biological parameters, *i.e*., LAI and chlorophyll content (the *R*^2^ values for these latter correlations were lower than 0.75). Therefore, these results indicate that it is feasible for us to consider WRT as a characteristic that reflects heavy metal stress levels.

### 4.2. Influence of Scaling on the Representative Characteristic (WRT)

**Figure 5:**
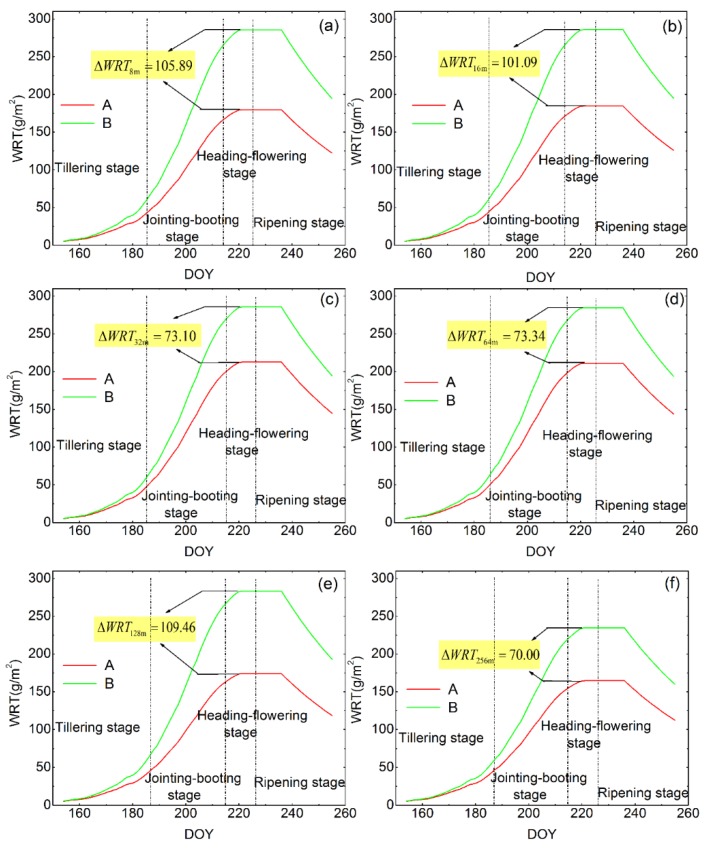
The effects of different spatial resolution (8 m, 16 m, 32 m, 64 m, 128 m, and 256 m) on WRT (dry weight of roots) estimation based on the RS-WOFOST framework of the two study areas.

As the WOFOST model was run pixel by pixel, we were able to obtain the WRT of all the rice pixels at each spatial scale. Figure 5 shows the mean simulated WRT curves for all the pixels in rice fields of each study area under different spatial scales; the averaged WRT curves reflect the general growth curves for the two stress levels and the effects of the scales on the simulated WRT. As shown in Figure 5, all the simulated WRT values presented a similar growth trend at the six scales. At the beginning of the rice growth period, the optimized WRT values were comparatively low and no significant differences between the two study areas were detected. When the rice growth entered into the jointing–booting stage, WRT values started to increase rapidly and the values of Area B were higher than those in Area A, thus indicating that there was a stress effect on the rice roots. The difference of WRT values between the two stress levels at each spatial scale during this period (DOY 171–DOY 220) are presented. As the spatial resolution degraded from 8 m to 16 m, the difference of WRT values showed a relatively minor decrease (Figure 5a,b), and then, a huge decline emerged at the spatial resolution of 32 m and 64 m while the difference was extremely small (Figure 5c,d). However, when the resolution was further degraded to 128 m, the difference of WRT changed drastically (Figure 5e). These results suggest that the critical threshold of the spatial resolution for investigating rice WRT during heavy metal stress monitoring was larger than 64 m but smaller than 256 m. Lastly, when the rice growth entered the heading–flowering stage (DOY 221–DOY 236), the WRT values of the two study areas maintained a constant maximum value for 16 days and the largest difference was observed between the two stress levels at the different spatial scale. This observation illustrates the effects of the spatial scale when evaluating heavy metal stress levels with simulated WRT values based on the assimilation of remote sensing data and the WOFOST model, *i.e*., different outcomes and conclusions were obtained at different spatial scales. Therefore, these types of effects should be considered when comparing the results from different studies.

**Figure 6:**
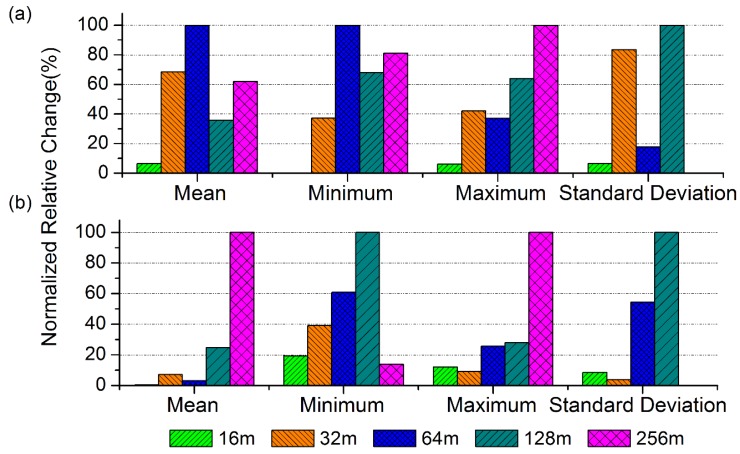
The normalized relative change of the mean, minimum, maximum, and standard deviation of WRT (dry weight of roots) as the spatial scale of LAI (leaf area index) information changed from 8 m to 16 m, 32 m, 64 m, 128 m, and 256 m in the two study areas: (**a**) Area A and (**b**) Area B.

In order to further analyze the influence of the spatial scale on the simulated WRT values, the statistical characteristics of the simulated rice WRT data for each spatial scale during a constant period of time (DOY 221–DOY 236) for all the rice pixels were calculated (Figure 6). As the spatial resolution upgraded from 8 m to 16 m, 32 m, 64 m, 128 m, and 256 m, the values of the mean simulated WRT in Area A changed from 179.53 to 184.61, 212.50, 211.12, 173.84, and 164.65, respectively. In contrast, the mean WRT values in Area B remained comparatively stable (normalized relative change within 8%) when the spatial resolution changed from 8 m to 64 m (Figure 6b). However, a large change was observed when the resolution was further degraded to 128 m, *i.e*., the normalized relative change suddenly increased to 24.7%. These results suggest that there is a critical spatial scale for data based on simulated WRT values. Moreover, it is worth noting that the change in Area A did not display a similar amount of variation.

With the purpose of further proving the existence of a critical threshold for monitoring heavy metal stress based on WRT values, the changes in statistical variability for WRT data (e.g., minimum and maximum values and standard deviations) at various spatial scales were evaluated. The change of WRT data was not dramatically influenced by the degradation of the spatial resolution from 8 m to 16 m in Area A (normalized relative change within 10% for all three statistics), whereas the change of WRT values in Area B was notable (19% for the minimum, 12% for the maximum). Then, the change gradually increased at the spatial resolutions of 32 m and 64 m in both areas and reached a maximum value when the spatial resolution coarsened to 128 m (values for the minimum and maximum WRT in Area A exceeded 60%) (Figure 6a). Overall, the variability metrics (*i.e*., minimum, maximum, and standard deviation) demonstrated significant changes (from 6% to 42%) in the WRT variability from 16 m to 64 m, and larger changes were observed in Area A when the resolution changed from 128 m to 256 m (60%–90%); the corresponding changes in Area B were relatively lower. These results indicate that a scale within 64 m to 128 m was the critical spatial threshold for adequately characterizing the WRT variability.

### 4.3. Identification of the Characteristic Scale

**Figure 7:**
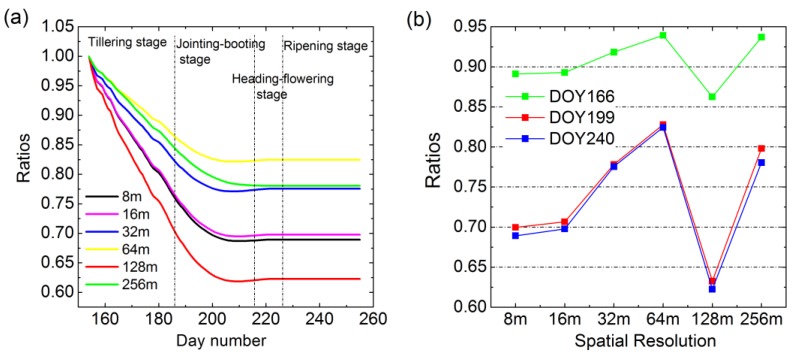
(**a**) Dynamic variations about the ratios of WRT (dry weight of roots) for Area A and Area B; and (**b**) the ratios of WRT (WRTA/WRTB) in the two study sites originating from the GF-1 (8 m) sensor and the aggregation of the GF-1 8 m image at coarser spatial resolutions (16 m, 32 m, 64 m, 128 m, 256 m) from various acquisition times (DOY 166, DOY 199, and DOY 240).

The effect of heavy metal stress was shown to be a dynamic process, which resulted in lasting changes in the WRT values. Compared to the quantitative method, the ratios could dynamically reflect the heavy metal stress levels on the rice root growth with a step-length of 1 d. As shown in Figure 7a, the ratios displayed a similar tendency at different scales throughout the entire growing season. At the beginning of the tillering stage, all ratios possessed the same relatively high value of 1, and then, the ratios presented a prominent descending trend where obvious differences were observed among the different scales. When the spatial resolution coarsened, the ratio decrease rates were slower than that at the original spatial resolution, but they did not show a regular declining tendency. The highest decrease rate was observed at the spatial resolution of 128 m, for which the ratio decreased down to 0.62 at the end of the tillering stage. The rate at which the ratios decreased with the day number (time series) can be explained by the different heavy metal stress levels. In these early stages, rice tissues had high demands for water and other nutrients, which are necessary for vegetative growth; meanwhile, toxic heavy metals along with other nutrient elements were being absorbed into the root cells. Therefore, the early growth stages can be considered to be a period in which heavy metal elements are accumulated. In the jointing–booting stage, the ratios continuously and noticeably declined, but the rate slowed down until it decreased to the minimum value. Meanwhile, the difference in the six varied spatial scales was more obvious, for example, the ratios could be distinguished at the spatial resolutions of 8 m and 16 m, whereas in the tillering stage, they were extremely similar. Thus, it can be concluded that the degree of heavy metal stress became more intense and reached its maximum when the plants entered into the jointing–booting stage. Then, the ratios increased slightly in the heading–flowering stage, and finally, all the ratios at different scales were relatively stable in the ripening stage. After entering the ripening stage, the rice plants along with the roots suffered from the aging process, which was associated with reduced absorptive capacities for water and nutrients. Therefore, the stress level remained roughly unchanged as the plant’s tolerance to heavy metals was stronger in these later stages.

The change of ratios describing the effects of scale on the dynamic assessment of heavy metal stress presented a similar trend at the different acquisition times (Figure 7b). At the spatial resolution of 8 m, the ratios remained low, and as the spatial resolution coarsened, the ratios started to increase. This increase in the ratios reflects the declining assessment efficiency for heavy metal stress. When the spatial resolution degraded to 64 m, the ratios presented a declining tendency until this reached the minimum value at the spatial resolution of 128 m, and then, the ratios increased again at the spatial resolution of 256 m; they actually became higher than those at the former spatial resolutions (8–64 m).

## 5. Discussion and Conclusions

In this study, the optimal characteristic scale for heavy metal stress monitoring in rice plants with remote sensing data was tentatively identified. Here, the WRT (*i.e*., the dry weight of roots) was consider as the representative characteristic, and this parameter has been shown to be a good indicator in previous studies [21]. Meanwhile, the assessment performance of WRT was favorably proven in terms of the sensitivity analysis that compared the results for WRT data to those for chlorophyll and LAI data. Thus, WRT appears to be a useful indicator for heavy metal stress in rice plants.

Importantly, increasing the spatial scale in LAI data derived from GF-1 imagery led to significant variation in the simulated WRT that could dynamically reflect the heavy metal stress levels, and thus, this contributed to the assessment efficiency. The relation between spatial scales and assessment performance for heavy metal stress found in this study is specific to the selected characteristic studied, *i.e*., WRT, and the results might differ for other characteristic parameters pursued or observed, other types of data used, and other retrieval and upscaling techniques; a similar conclusion was presented by Garrigues *et al.* [46] in relation to the definition of the optimal scale. Nevertheless, the dramatic change of the spatial scale from 64 m to 128 m for the difference in WRT between the two study areas can reasonably be extrapolated to the critical spatial scale. A ratio analysis of the WRT values showed variable decreasing rates under different spatial resolutions and the highest one was observed at 128 m. The results obtained could be explained by the increase of the efficiency assessment. According to the study by Quattrochi *et al.* [13], characteristic scales define the space and time intervals with which a process can be detected and monitored. The results presented in the current study suggest that the scale of 128 m can be considered as the turning point in heavy metal stress assessments and could be deemed as the upper limit for effective monitoring. Beyond the characteristic scale, the change in the simulated WRT and corresponding ratios became unpredictable. The critical threshold value of 128 m could be pervasive because the agriculture monitoring occur at or below this spatial scale in most cases.

However, one issue should be addressed properly about the general problem of heavy metal stress monitoring. Is it still effective when we applied the threshold of 128 m found in this study to a large area or other crops? The model parameters and climate parameters of the WOFOST model could be adjusted to simulate the growth of various crops around the world [47]. Thus, when apply the RS-WOFOST assimilation framework to a large area or other crops, we should firstly calibrate the related parameters to the actual condition. Furthermore, the ratio analysis presented in this study ought to be proved. In conclusion, the generality of this finding in other areas and crops are supposed to be further confirmed.

Considering the reduction of data costs and improvements in monitoring accuracy, the identification of the characteristic scale has an important significance for effective and reliable rice heavy metal stress monitoring. Furthermore, the analysis presented here also concerns the problem of selecting the appropriate spatial scale for a given application. The results of the ratio analysis clearly indicate that the choice of a spatial scale should be made on the basis of the characteristic of the object of interest and they provide an analytic framework in terms of properly identifying the appropriate scale to observe heavy metal stress from remote sensing imagery.

Our results are limited by the selected characteristic’s sensitivity to the heavy metal stress and the upscaling approaches used, but the maximum of the 128 m value identified via the statistical properties and the ratio analyses is an important indication of the sufficient spatial resolution needed to fully investigate heavy metal stress levels based on WRT assimilated by remote sensing data and the WOFOST model. Meanwhile, it should be pointed out that the characteristic scale was qualitatively analyzed in this study, and the performance of the characteristic scale could be further investigated quantitatively through the use of measured data and more detailed procedures. The errors in RS-WOFOST model simulations caused by different upscaling methods represent another issue that should be addressed in future studies.

## Figures and Tables

**Table 1:** The mean red soil of top layer and rice heavy metal concentrations in the two study areas.

Heavy Metals	Background Value (bi) ^1^	A			B		
		(113°06′E, 27°45′N)			(113°14′E, 27°37′N)		
		Soil (si)	Rice Tissue ^2^	Pollution Index (si/bi)	Soil (ci)	Rice Tissue ^2^	Pollution Index (si/bi)
Cd	1.43	3.27	5.9	2.29	2.25	3.23	1.57
Hg	0.2	0.51	0.06	2.55	0.29	0.04	1.45
Pb	82.78	109.93	36.73	1.33	89.67	15.18	1.08
As	19.11	18.15	7.04	0.95	18.33	6.29	0.96
Pollution Level		Level II (Serve stress)			Level I (Light stress)		

Note: ^1^ Background values of heavy metals were derived from the Hunan Institute of Geophysical and Geochemical Exploration, China; ^2^ The rice concentration of heavy metals in the two sampling plots were the mean values of *in situ* measured three important rice phenological stages (tillering stage (mid-June), jointing-booting stage (mid-July) and ripening stage (late August)).
